# CD19(+) B Cells Confer Protection against Experimental Cerebral Malaria in Semi-Immune Rodent Model

**DOI:** 10.1371/journal.pone.0064836

**Published:** 2013-05-28

**Authors:** Lam Quoc Bao, Nguyen Tien Huy, Mihoko Kikuchi, Tetsuo Yanagi, Masachika Senba, Mohammed Nasir Shuaibu, Kiri Honma, Katsuyuki Yui, Kenji Hirayama

**Affiliations:** 1 Department of Immunogenetics, Institute of Tropical Medicine, Nagasaki University, Nagasaki, Japan; 2 Center for International Collaborative Research, Nagasaki University, Nagasaki, Japan; 3 Animal Research Center for Tropical Infections, Institute of Tropical Medicine, Nagasaki University, Nagasaki, Japan; 4 Department of Pathology, Institute of Tropical Medicine, Nagasaki University, Nagasaki, Japan; 5 Department of Molecular Microbiology and Immunology, Graduate School of Biomedical Sciences, Nagasaki University, Nagasaki, Japan; 6 Global Center of Excellence (GCOE), Nagasaki University, Nagasaki, Japan; Obihiro University of Agriculture and Veterinary Medicine, United States of America

## Abstract

In African endemic area, adults are less vulnerable to cerebral malaria than children probably because of acquired partial immunity or semi-immune status. Here, we developed an experimental cerebral malaria (ECM) model for semi-immune mice. C57BL/6 (B6) mice underwent one, two and three cycles of infection and radical treatment (1-cure, 2-cure and 3-cure, respectively) before being finally challenged with 10^4^ *Plasmodium berghei ANKA* without treatment. Our results showed that 100% of naïve (0-cure), 67% of 1-cure, 37% of 2-cure and none of 3-cure mice succumbed to ECM within 10 days post challenge infection. In the protected 3-cure mice, significantly higher levels of plasma IL-10 and lower levels of IFN-γ than the others on day 7 post challenge infection were observed. Major increased lymphocyte subset of IL-10 positive cells in 3-cure mice was CD5(−)CD19(+) B cells. Passive transfer of splenic CD19(+) cells from 3-cure mice protected naïve mice from ECM. Additionally, aged 3-cure mice were also protected from ECM 12 and 20 months after the last challenge infection. In conclusion, mice became completely resistant to ECM after three exposures to malaria. CD19(+) B cells are determinants in protective mechanism of semi-immune mice against ECM possibly via modulatory IL-10 for pathogenic IFN-γ production.

## Background

Malaria caused an estimated 655,000 deaths in 2010, mostly among African children [1]. In sub-Saharan Africa, malaria might account for 40% of pediatric admissions to some hospitals, 10% of which may be due to cerebral malaria [2]. The prevalence of cerebral malaria in endemic areas in Africa (Zambia, Kenya, Tanzania and Malawi) was 1.12 cases per/1000 children per year [3]. Mortality from cerebral malaria remained between 10% and 14% in sub-Saharan and Southeast Asia [4]–[6].

In high transmission areas, the adults, as semi-immune individuals, were less vulnerable to cerebral malaria than children because of acquired partial immunity [7], [8]. The incidence rate of cerebral malaria in patients aged from 16 years and above was less than 10% as compared to 34% of patients aged under 5 years [7]. Naturally acquired immunity to malaria minimizes malaria morbidity and mortality in older children and adults living in intensive *Plasmodium sp*. transmission regions [9], [10]. Noticeably, cerebral malaria rarely occurs in very young children at their first malaria infection, but often attacks young children undergoing second or subsequent malaria infections [11], [12]. However, the mechanisms leading to protection versus death from cerebral malaria and why cerebral malaria was more common in children than in adults [13], [14] are not yet fully understood. It has been proposed that T cells but not antibody response induced the immunity against malaria in human experiment of repeated malaria infection [15]. Immunomodulatory mechanism was involved in preventing ECM in mouse model [16]. Recently, IL-10 producing regulatory CD19(+) B cells has been intensively studied in abrogating immune-pathologies [17]–[19] and modulatory IL-10 has been beneficial for prevention of auto-immune diseases [20]–[23].

In mouse model, it is known that C57BL/6 mice are susceptible to ECM induced by virulent *Plasmodium berghei ANKA* (*Pb*A) [14], [24] and usually die from typical neurological symptoms between days 6–10 after infection [25]–[28]. Approximately, 95% to 100% of those mice exhibited the symptoms of ECM such as coma, convulsion, paralysis and hemiplegia, and died within 14 days of infection [29]–[31]. The C57BL/6 mouse model was well-established to identify the host determinants in protective immunity and malaria parasite mediated immunopathology [32], [33]. Previously, we have established a semi-immune malaria model using *Pb*A and C57BL/6 mice delineating the mechanism of destruction of erythrocytes and severe malaria anemia [34], [35], but ECM was not mentioned. Here, we asked the question whether the semi-immune could prevent the development of ECM in the *Pb*A-infected C57BL/6 model, and what are the factors in this regulatory and protective mechanism. We demonstrated that three exposures to malaria infection provided the resistance to ECM. Our data indicated the involvement of CD19(+) cells in the protection of ECM in semi-immune model.

## Results

### Three cycles of malaria infection and treatment are necessary for mice to become completely resistant to cerebral malaria

**Figure 1:**
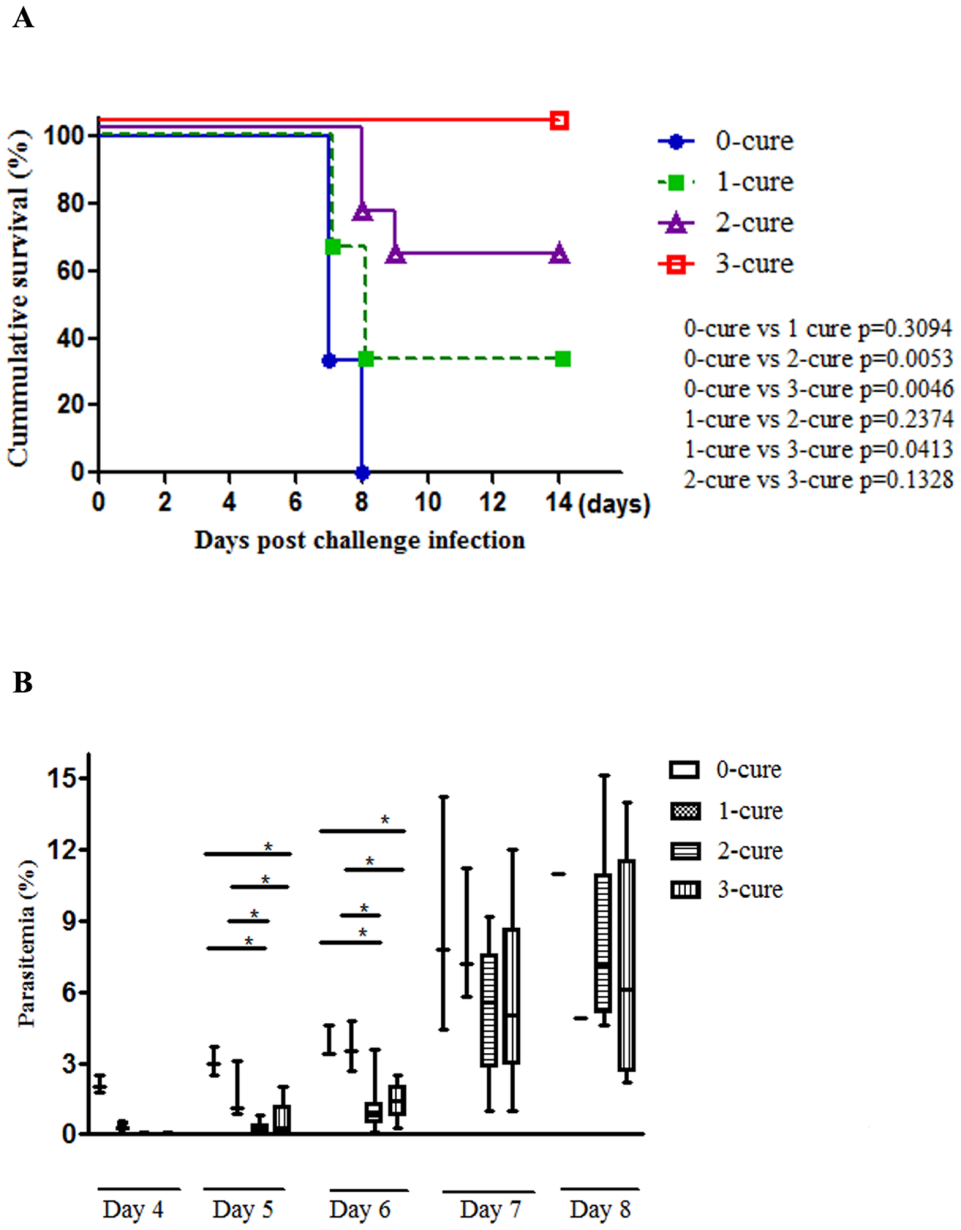
Three cycles of infection and cure are critical for the absolute protection against ECM. All mice in the four groups were finally challenged with 10^4^ *Pb*A-pRBCs without treatment. Survival and parasitemia were monitored daily. (**A**) Survival curve of the different cure group mice. Three-cure mice were completely resistant and had significantly improved survival rates compared with the others. Statistical significance was determined by log-rank test. (**B**) Median parasitemia (%) of mice in all groups over the time of infection. Parasitemia (%) of these groups of mice was performed on days post challenge as indicated and was shown by the box-plots. The upper and lower limits of the box are corresponding to the 25^th^ and 75^th^ percentile and the horizontal line inside the box is the median. The whiskers extend to the minimum and maximum values that data assume. Comparisons between each two groups of mice on each day was statistically analyzed by using the nonparametric Mann-Whitney U test, where *, p<0.05. Data shown are representative of two independent experiments; 0-cure mice, n = 3; 1-cure mice, n = 3; 2-cure mice, n = 8; 3-cure mice, n = 5.

Semi-immune mice were generated by performing 0, 1, 2, and 3 cycles of infection and radical anti-malaria treatment, namely 0-cure, 1-cure, 2-cure, and 3-cure mice. We found that within 7–9 days post challenge infection (PI), 100% of 0-cure mice, 67% of 1-cure mice and 37% of 2-cure mice (Fig.1A) succumbed to cerebral malaria with neurological signs including hemi-paresis, ataxia, paralysis, seizures and coma. In addition, the post-mortem brain histopathology images showed vascular wall disruption, hemorrhage, and sequestration of infected erythrocytes (Fig.S1). Conversely, 100% of infected 3-cure mice survived beyond day 14 and did not develop ECM. There was no reduction in mortality caused by ECM between 0- and 1-cure mice (p = 0.3094, log-rank test), whereas the survival rate noticeably increased among 2-cure mice compared to 0-cure mice (p = 0.0053, log-rank test). However, two cycles of *Pb*A infection and anti-malaria treatment were not sufficient to produce an optimal immune status to protect mice completely from ECM. The survival rate was significantly higher in 3-cure mice compared with 0-cure controls (p = 0.0046, log-rank test). The result was reproduced in another independent experiment. Thus, three infection-cure rounds were needed to establish the ECM-resistant mice. Additionally, the frequency of resistance in ECM-susceptible B6 mice increased dramatically after each cycle of infection and treatment (Fig.1A), suggesting that protective mechanism was further remarkably enhanced after each one.

Next, we examined the parasitemia of all mice in the different cure groups. Parasitemia increased steadily in the 0- and 1-cure mice as well as in the 2- and 3-cure mice (Fig.1B). However, highly ECM-resistant mice, including 2- and 3-cure mice, had significantly lower parasitemia during the first six days of infection, as indicated on day 4, 5 and 6 (p<0.05; Fig.1B), showing that the mice exposed to more cycle of infection were able to control parasitemia more efficiently than 0- and 1-cure.

### Inhibition of ECM development in 3-cure mice was not due to the reduction in parasitemia level during the early stage of infection

**Figure 2:**
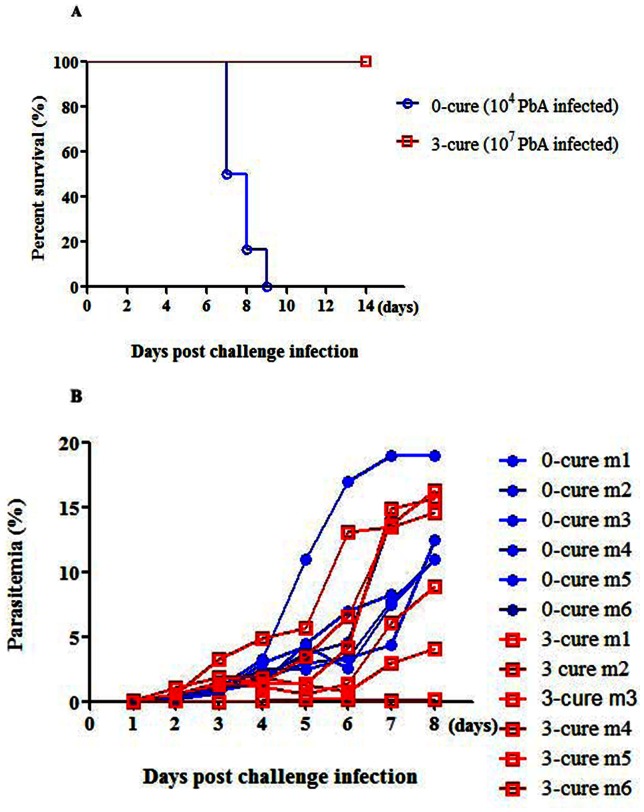
High dose of *Pb* ANKA (10^7^ *Pb*A-pRBCs per mouse) could not induce ECM in 3-cure mice. Three-cure mice were challenged with high dose of *Pb*A (10^7^ *Pb*A-pRBCs per mouse) as compared to 0-cure mice inoculated with normal dose (10^4^ *Pb*A-pRBCs per mouse). After infection, all mice were daily monitored for survival and parasitemia. (**A**) Survival curves. Statistical analysis was performed by log-rank test, p<0.0002. (**B**) Kinetics of individual parasitemia of 3-cure and 0-cure mice. Comparisons between two groups of mice on each day was statistically analyzed by Mann-Whitney U test, where NS, p>0.05. n = 6 mice in each group.

Since parasitemia levels were inhibited during early period of infection, we examine the possibility that 3-cure protection was derived from lowered peripheral parasite. We generated high parasitemia in 3-cure mice by challenging them with high dose (10^7^) of *Pb*A-pRBCs while 0-cure mice were inoculated with 10^4^ *PbA*-pRBCs (as the control). Although all of 3-cure mice infected with higher *Pb*A-pRBCs dose showed comparable parasitemia level to the 0-cure controls, the high-dose *Pb*A-pRBCs infected mice did not develop ECM (Fig.2A, B). Significant difference in survival between 0- and 3-cure mice was recorded over the course of infection (p = 0.0002, using log-rank test for survival (p>0.05, Fig.2A). Additionally, similarity in parasitemia was observed between two mice-groups throughout the first week of infection (p>0.05, Fig.2B), clearly demonstrating that ECM developed in 0-cure mice at higher parasitemia levels at which ECM did not occur in 3-cure counterparts. Alternatively, parasitemia was not correlated to ECM-resistance in 3-cure mice. Taken these data together, we concluded that there was an alternative protective mechanism other than lowered-parasitemia in 3-cure mice.

### *Pb*A-specific IgG antibody levels day 7 PI in ECM-resistant and susceptible mice

**Figure 3:**
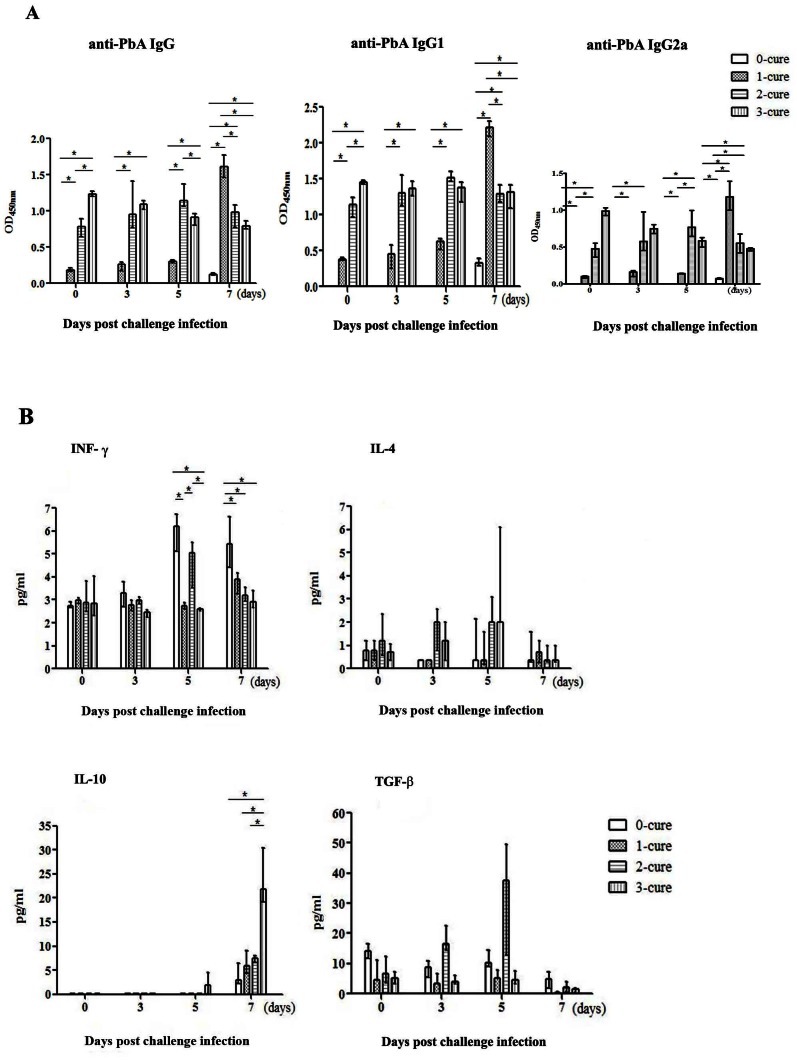
Persistent parasite-specific antibodies and sharp increase of IL-10 production on day 7 PI in 3-cure. Plasma antibodies and cytokines were quantified on day 0, 3, 5, and 7 post challenge infection (PI). On each of indicated days PI, four mice in each group were sacrificed to harvest sera for antibody and cytokine quantitation. Bar chart represents the median antibody and cytokine levels (n = 4 on each group) with the error bars indicate the interquartile range (lower bar: 25^th^ percentile, upper bar: 75^th^ percentile). (**A**) IgG, IgG1, IgG2a; (**B**) IFN-γ, IL-4, IL-10 and TGF-β levels in plasma during the first week of infection. Data presented are pooled from two separate experiments. Statistical analysis was by Mann-Whitney U test comparing the differences on the indicated days PI between 0-cure and each of 1-cure, 2-cure and 3-cure mice where *, p<0.05.

To investigate the humoral immune response in semi-immune mice, we initially assessed the role of malaria-specific antibody. As shown, from day 0 to day 5 PI, the levels of all IgG, IgG1 and IgG2a in 2 and 3-cure mice were significantly higher than in 0- and 1-cure mice (p<0.05, using Mann-Whitney U test) (Fig.3A). Although on day 7, all types of malaria-specific antibody drastically increased in 1-cure mice whereas 2- and 3-cure mice did not show any change (Fig.3A), most of the ECM-susceptible mice (1-cure) became moribund around this day. The significantly higher antibody amounts in 1-cure on day 7 PI indicate the normal booster response whereas 2- and 3-cure mice antibody response was marginal or even suppressed. There was no significant difference in antibody responses (Fig.3A) between 2-cure and 3-cure mice. Although not statistically significant, survival rate was slightly lower in 2-cure mice (63%) than 3-cure mice (100%) (p = 0.1328) (Fig.1A), suggesting that malaria specific antibodies could not provide 100% of protection. Further study with larger number of mice will confirm this issue. Vigorous humoral immune response in 1-cure mice at late stage of ECM could not rescue them from ECM.

### Plasma level of IFN-γ was suppressed on day 5 PI and that of IL-10 vigorously increased on day 7 PI in ECM-resistant mice

ECM has been suggested as the result of excessive immune responses leading to overproduction of pro-inflammatory cytokines in which IFN-**γ** is essential in ECM pathogenesis. To look for the possible suppression to immune responses in ECM-resistant mice, we further assess Th1, Th2 activation and immuno-modulators in mice with different degrees of immunity. We examined profile of IFN-**γ**, a pathogenic Th1 cytokine of ECM [36]–[38], IL-4, a Th2 cytokine, TGF-β and IL-10, regulatory cytokines [39] during the first week of infection. The amount of IFN-γ was similar in all groups on day 0 PI, increased and became higher in 0- and 1-cure, but suppressed in 2- and 3-cure mice on day 7 PI (p = 0.021, except for 1-cure vs. 2-cure with p = 0.149) (Fig.3B). IL-4 levels were similarly low in all groups during the course of observation, slightly increased on day 5 PI and also inhibited on day 7 PI in 2 and 3-cure mice (Fig.3B). Together, Th1 and Th2 cytokines were suppressed in ECM-resistant mice on day 7 PI. Active TGF-β, a regulatory cytokine, was present at low levels and appeared to be negatively correlated with IL-10 on day 7 PI in all groups of mice (Fig.3B). By contrast, IL-10 levels started increasing on day 5 PI in all mice-groups, but dramatically elevated in 3-cure mice on day 7 compared to the others (p = 0.021) (Fig.3B). There was a gradual increase of IL-10 levels from 0- to 2-cure mice but a big jump in 3-cure mice around day 7, implying the enhancement of IL-10 production after each cycle of malaria infection and treatment. Remarkably, higher level of plasma IL-10 in ECM resistant 3-cure mice versus its much lower level in 0-, 1-, and 2-cure mice suggested that the inhibition of exacerbated inflammatory responses was possibly via IL-10 in 3-cure mice.

### CD19(+) B cells are a major source of IL-10, in which IL-10(+)CD5(−)CD19(+) cells appear as the main subset

**Figure 4:**
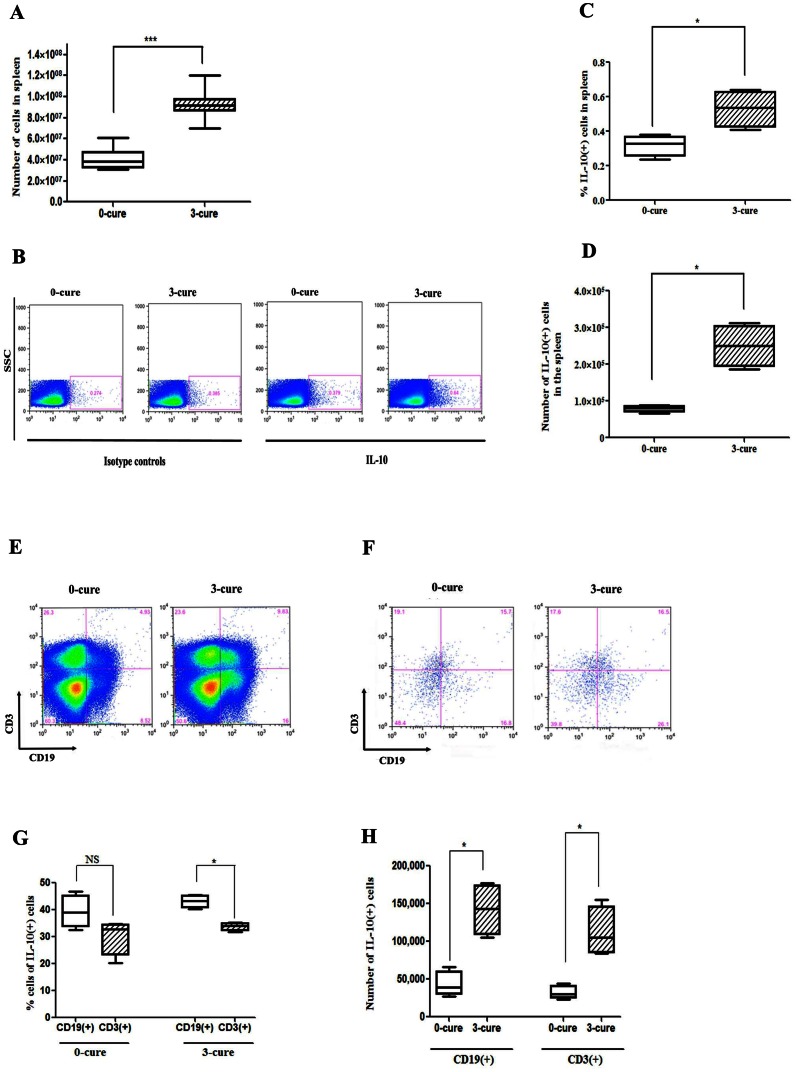
CD19(+) cells are the major source of IL-10. Spleen cells from infected B6 mice were harvested and stimulated for 5 hours in culture with pRBC, LPS, PMA, ionomycine and monensin, which was followed by IL-10 intracellular staining and analyzed by flow cytometric analysis. (**A**) Bar chart shows the median number of splenocytes (± interquartile range) (n = 10). (**B**) Representative dot plots for one mouse of each group showing frequencies of IL-10 producing cells in spleen. (**C, D**) Bar charts indicate the median (± interquartile range) percentages and numbers of cells that produced IL-10 (n = 4). (**E, F**) Representative dot plots for one mouse of each group showing the proportion of CD19(+) and CD3(+) cells before and after gating for IL-10(+) cells. (**G, H**) Bar charts represent median (± interquartile range) percentages (**G**) and numbers (**H**) of CD19(+)IL-10(+) and CD3(+)IL-10(+) in IL10(+) cells in spleen. All results represent two independent experiments with four mice in each group. Significant differences between sample medians are indicated; *, p<0.05; ^***^, p<0.001; NS, non-significant by Mann-Whitney U test.

We further sought the major source of IL-10 in the spleen of ECM-susceptible, 0-cure and ECM-resistant, 3-cure mice. The median of total splenocytes' numbers of day 7 PI 3-cure mice (9.17×10^7^) was 2.2 fold higher than those of 0-cure mice (3.81×10^7^), indicating that repeated exposure of malaria enhanced the proliferation of splenocytes (Fig.4A). Because the increased IL-10 level in the plasma was suggested to be related to the protection of ECM in 3-cure mice, we investigated IL-10 producing spleen cells of 3-cure mice. Splenocytes were collected from 0-cure and 3-cure mice on day 7 PI and were cultured with parasitized erythrocytes, lipopolysaccharides (LPS), phorbol 12-myristate 13-acetate (PMA), ionomycin, and monensin (Golgi plug) for 5 hours. Intracellular cytokine staining revealed that the median percentage and absolute number of IL-10(+) cells were 1.94-fold and 3.62-fold higher in 3-cure (0.97% and 726,930) than those in 0-cure mice (0.5% and 200,421) on day 7 PI, respectively (Fig.4B-D, p<0.05). Next, we determined the contribution of B cells and T cells in IL-10 production in 3-cure mice and 0-cure controls by flow cytometric analysis. CD19(+) is a B cell-specific surface molecule that defines signaling thresholds critical for B-cell responses and autoimmunity [40]. In 3-cure mice, IL-10(+)CD19(+) B cells accounted for 52.9% of IL-10(+) cells; which was 2.1-fold and 1.7-fold higher in proportion and absolute number compared to IL-10(+)CD3(+) cells, respectively (Fig.4F-H, p<0.05). On the other hand, although not significant, IL-10 was expressed at higher amount in CD19(+) B cells (51.81%) than in CD3(+) cells (33.35%) in 0-cure mice. Median numbers of both IL-10(+)CD19(+) and IL-10(+)CD3(+) cells were increased more than 4-fold in 3-cure mice compared to 0-cure controls (Fig.4H, p<0.05). CD19(+)CD3(+) cells present in spleen of both 0-cure and 3-cure mice (Fig.4E, 4F) are possibly immature B cells [41]. This hypothesis would be compatible with the observation that progenitor cells were increased in peripheral blood of children with malaria [42].

**Figure 5:**
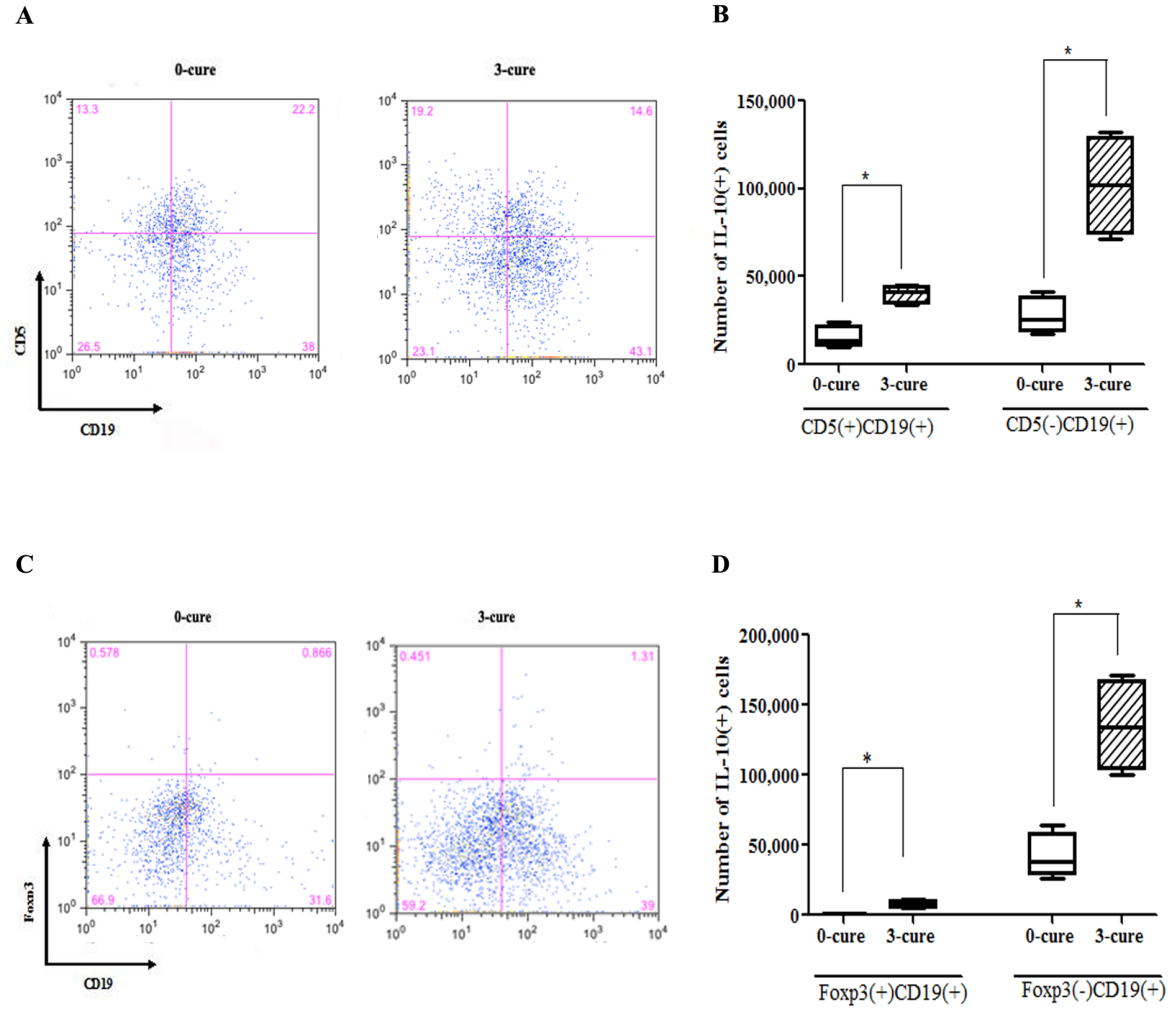
B cell subset CD5(−)CD19(+) (B1b) predominantly produces IL-10. Splenocytes from day 7 PI 0- and 3-cure mice were cultured with pRBC, LPS, PMA, ionomycin, and monensin for 5 hours before permeabilization and staining with CD5, CD19, Foxp3 and IL-10 mAbs and analyzed by flow cytometric analysis. (**A, C**) Representative dot plots for a mouse from each group show the frequencies of CD5(**A**) and Foxp3(**C**) expression in IL-10 producing CD19(+) cells. (**B, D**) Bar graphs indicate the median (± interquartile range) numbers of CD5(+), CD5(−) cells (**B**) and Foxp3(+), Foxp3(−) cells (**D**) in IL-10 producing CD19(+) cells. All results are from two independent experiments with four mice in each group. Significant differences between sample medians are indicated; *, p<0.05, by Mann-Whitney U test.

Several subsets of IL-10(+)CD19(+) B cells have been intensively reported as immune-suppressors in other diseases including arthritis, experimental autoimmune encephalomyelitis [19], [17], [18]. CD5 is a pan-T cell surface marker that is also present on regulatory and memory B cells [43], [18], [44]. Previously, B10 (CD1d^high^CD5+CD19+) cells, B1a (CD5+CD19+) cells produced high amount of IL-10 [18], [45] and regulatory B cells (Foxp3+CD5+CD19+ B cells) were present in human peripheral blood mononuclear cells [46]. Thus, we analyzed the expression of CD5 and Foxp3 in IL-10(+)CD19(+) B cells. The result showed that among the IL-10(+)CD19(+) B cells (Fig.4H), numbers of CD5(−) B cells was 4.4 and 4.2-fold higher than those of CD5(+) B cells in 0- and 3-cure mice, respectively (Fig.5A, B). Although accounting for low distribution (Fig.5C, D), the number of Foxp3(+)IL-10(+)CD19(+) B cells was significantly increased in 3-cure mice compared to 0-cure counterparts (Fig.5C, D).

### Naïve B6 mice receiving CD19(+) B cells transfer from 3-cure mice were resistant against ECM

**Figure 6:**
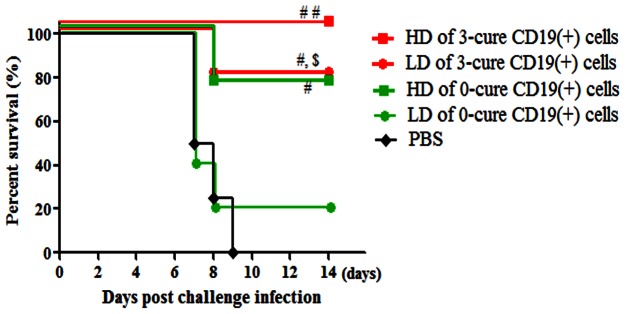
CD19(+) splenocytes of 3-cure mice provided complete resistance against ECM to B6 naïve mice. Splenocytes from each of day 7-infected 0-cure or 3-cure mice were separated into CD11c positive and negative populations. CD11c(−) cells were then sorted by anti-CD19 magnetic beads into purified CD19(+) fraction, which was adoptively transferred into naïve B6 mice by i.v. tail inoculation. Recipient mice were challenged with 10^4^ *Pb*A 24 hours after transfer. Curves are survival of mice that received high dose (HD) (3×10^7^) and low dose (LD) (1.5×10^7^) of 0-cure or 3-cure CD19(+) cells per mouse. The purity of the CD19(+) fraction was >90%. Data presented are representative of two independent experiments with four to five mice per group. Significant differences between mouse groupsare indicated using log-rank test, where #p<0.05 and ##p<0.01 versus PBS; $p<0.05 versus LD of 0-cure CD19(+) cells.

To test the hypothesis that CD19(+) B cells derived from 3-cure mice splenocytes may confer the abrogation of immune-mediated pathology, CD19(+) B cells in spleen were isolated and passively transferred to naive mice. After first separation of CD11c(−) splenocytes of 3-cure and 0-cure mice at day 7 PI, we then sorted CD11c(-) cells based on antibody to CD19 conjugated magnetic beads to isolate CD19(+) cells. Before transfer experiment, purity of CD19(+) cells was assessed. The average cell numbers of CD19(+) cells were 3×10^7^ and 1.5×10^7^ in one spleen of 3-cure and 0-cure mice, respectively. The obtained CD19(+) cells were then intravenously injected at high dose (3×10^7^ cells per mouse) and low dose (1.5×10^7^ cells per mouse) into naïve mice followed by intra-peritoneal infection of 10^4^ *Pb*A-pRBCs. As shown in Fig.6, 100% and 80% of mice receiving high and low dose of 3-cure CD19(+) cells, respectively survived compared with 0% of survival in PBS treated controls, indicating that CD19(+) cells from 3-cure mice controlled the pathology. On the contrary, in mice treated with low dose of 0-cure CD19(+) cells, survival rate was much reduced, only 20% (Fig.6), revealing that CD19(+) cells of 0-cure mice were insufficient to inhibit ECM. However, transfer of 0-cure CD19(+) cells at high dose improved survival rate of recipient mice to 75%, suggesting dose-dependent protection of 0-cure CD19(+) cells.

### Protection against ECM in semi-immune mice existed throughout life

**Figure 7:**
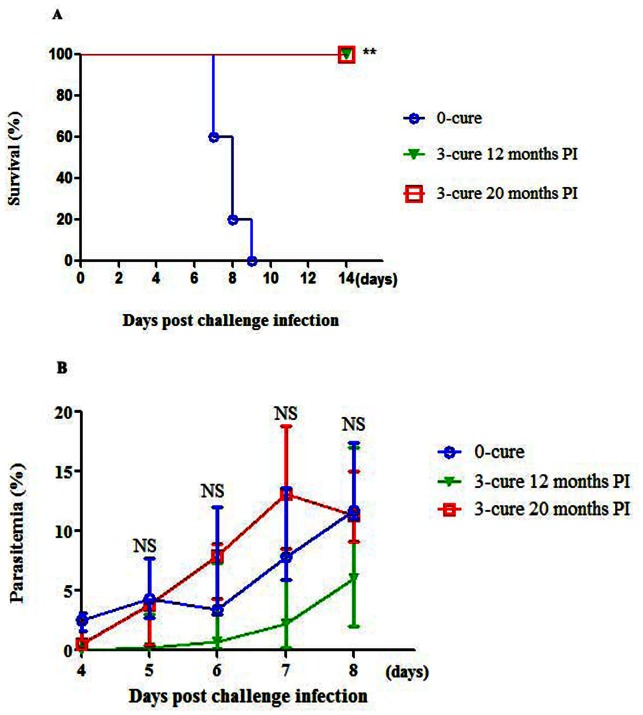
Long-lived resistance against ECM in 3-cure mice. After challenge infection, 3-cure mice fully recovered with clearance of parasitemia around day 21 PI. They were regularly examined at 3-month intervals and showed no parasitemia. Twelve and twenty months post the last challenge infection, these aged 3-cure mice were re-challenged with 10^4^ of homologous *Pb*A-pRBCs. The age-matched B6 mice were used as control. (**A**) Survival curves and (**B**) Parasitemia profile. Data presented are from one experiment with three to five mice in each group. Statistic significance between 0-cure and 3-cure was performed by using log-rank test and Mann-Whitney U test, where NS, p>0.05; **, p<0.01.

For understanding of the longevity of protection against ECM in the absence of persisting exposure, 3-cure mice were re-challenged with 10^4^ *Pb*A-pRBCs twelve and twenty months after the last challenge. It was recorded that 100% of the aged mice were still resistant to ECM compared with 100% ECM-induced death of age-matched 0-cure counterparts (Fig.7A, p<0.01). No significant difference in parasitemia was observed between aged 3-cure mice and 0-cure controls during the first week of infection (Fig.7B), once again indicating a resistant mechanism independent to parasitemia.

## Discussion

In this work, we established a semi-immune model in ECM-susceptible mouse strain with virulent *PbA*. This was achieved by repeated infection and radical anti-malaria treatment, mimicking the natural boost infection and recovery in humans in areas of intense *P. falciparum* transmission. We found an absolute protection against ECM in 3-cure mice. Simultaneously, we also demonstrated that CD19(+) B cells of the semi-immune mice promote their acquired immunity to ECM.

Splenic CD19(+) B cells, including regulatory and memory B cells [44], [18], of infected 3-cure mice were proved to completely protect naïve B6 mice against ECM in the passive transfer experiments, confirming the beneficial effects of CD19(+) cells in pathology. High survival rate (75%) of mice transferred with CD19(+) cells from two spleens of infected 0-cure mice highlighted that protective CD19(+) cells were present in 0-cure mice but their number was not enough to prevent ECM.

Apart from the correlation with immunopathogenesis in autoimmune disease [47], understanding of CD19(+) is still un-clarified in infectious disease, particularly in ECM. CD19(+) cells have been known to play a protective role in the control of pathogenic T cell activation in previous studies [18]. It was found that CD19 (−/−) mice had augmented experimental autoimmune encephalomyelitis responses and develop severe non-remitting form of the disease [48]. In addition, similar benefits of CD19(+) were evident in CD19(−/−) mice which had increased contact hypersensitivity responses after subsequent contact with the sensitizing antigen [49]. Our data were in consistent with these findings that CD19(+) cells enhanced the protective immunity against the immune-pathologic diseases including ECM.

IL-10 appears to oppose the immunopathological process [50]–[53] which is driven by IFN-γ, a key pathogenic factor of ECM [37], [36], [38]. It was discovered that three exposures to malaria increased mouse's splenocytes number drastically compared to 0-cure counterparts. The abrupt increase of IL-10(+) cells in splenocytes of 3-cure mice than that in 0-cure controls, could account for the lack of ECM in 3-cure mice via the augmentation of peripheral IL-10 concentration. The in-vitro experiments revealed that CD19(+) B cells in spleen of 3-cure mice were the major source of IL-10 upon 5 hour-stimulation. So far, no study has been reported on protective regulatory B cells in ECM. Besides, CD3(+) cells expressed a considerable amount of IL-10, but significantly less than CD19(+) B cells. IL-10(+)CD19(+) B cells and IL-10(+)CD3(+) T cells in infected spleen were significantly increased in number after being exposed to malaria three times compared to infected 0-cure controls.

Regarding the subset of IL-10(+)CD19(+) B cells, majority of these cells were negative in CD5 and Foxp3 expression. As shown, Foxp3(+)IL-10(+)CD19(+) cells accounted for a low percentage of IL-10(+) cells but significantly higher in 3-cure than 0-cure mice. Regulatory B cells, including Foxp3(+)CD5(+)CD19(+) and regulatory CD1d^hi^CD5(+)CD19(+) B cells (B1a) have been intensively studied in recent years [54]–[61], [19], [18]. TLR-mediated negative modulation of B cell IL-10 evolved as a mechanism that prevents an exacerbation of acquired antigen-specific responses (T cell responses) [17]. IL-10 secreted from these B cells could then down-regulate the local immune responses by a ‘negative-feedback loop’ resulting in the limitation of excessive self-tissue injuries [17]. Nonetheless, CD5(−)CD19(+) cells (B1b cells) were pivotally responsible for IL-10 production in 3-cure mice. Our results diverged from studies which suggested B1a cells as the major IL-10 secreting cells [18]. IL-10 production by Foxp3(+)CD19(+) have not been mentioned previously [62]. It appeared to be the first time Foxp3(+)IL-10(+)CD19(+) cells were identified as a new subset of IL-10 producing B cells in our mouse model study. This minor population will be further investigated for the role of immune-modulation. Collectively, CD19(+) B cells prevented ECM in naïve mice possibly via regulatory IL-10 derived mostly from B1b cells. The second source of IL-10 in 3-cure mice was CD3(+) cells which were considered as the predominant IL-10 producers in infected naïve B6 mice in previous malaria studies [63], [64]. In line with one of our findings (data not shown), Th1 cells were reported as the source of IL-10 in *Plasmodium chabaudi chabaudi* AS infection to protect mice from pathology during acute phase [65]. Additionally, macrophages, mast cells, NK cells, neutrophils and eosinophils were also reported as origins of regulatory IL-10 [66], [62], [65], [67], [68]. The method used here for stimulating IL-10 secreting cells is cocktail of parasitized red blood cells (pRBC), lipopolysacharide (LPS), phorbol 12-myristate 13-acetate (PMA), ionomycin. This method was modified from previous studies [18], [69]. Neither pRBC nor cocktail of LPS, PMA and ionomycin resulted in stimulation of IL-10. It may be attributed to the differences between in vitro and in vivo condition. Using IL-10 reporter mice could be better for identification of IL-10 production from all myeloid and lymphoid cell types [70].

In 3-cure mice on day 7 PI, profoundly high plasma levels of IL-10 and suppressed levels of IFN-γ, known to play a key role in the development of ECM [71], [72], implied that IL-10 was a much potential modulator in this protection. Of notes, activation of Th1 cytokines including IFN-γ was modulated by IL-10 [73], [52], [53]. It was previously found that both IFN-γ and IFN-γ receptor-deficient mice showed resistance to the development of ECM [74], [75] while IL-10 has been strongly assumed as immune-modulator [76]-[78], [68], [79]. Previously, filaria-infected B6 mice was protected from full progression to ECM via suppressing the overwhelming inflammatory reaction by IL-10 [80]. 40% of Balb/c ECM-resistant mice developed ECM when treated with IL-10 neutralizing antibody and 70% of recombinant IL-10 treated ECM-susceptible CBA mice were protected from ECM [81]. Accordingly, IL-10, mostly from CD19(+) B cells, could be a defense barrier for 3-cure mice against ECM.

Reducing ECM in naïve mice via low parasitemia has been reported in *Pb*A infected C57BL/6 mouse model, in which the parasitemia was correlated with reduced levels of pathogenic cytokines, chemokines, CD8+ T cells and parasites in the brain [28], [82], [83]. In contrast, other studies have reported that parasite burden does not appear to play a pivotal role in the development of ECM in the *Pb*A model [84]–[86], [16], [87]. It was reported that malaria specific antibodies inhibited parasite proliferation [88]. In our study, 3-cure mice, having lower parasitemia and higher malaria specific antibodies than 0-cure counterparts, did not develop ECM during the course of infection. However, high dose of *Pb*A infection in 3-cure mice induced parasitemia similar to 0-cure controls but none of 3-cure mice manifested symptoms of ECM, demonstrating that parasitemia-independent factors contributed to ECM resistance in semi-immune mice. Instead, regulatory IL-10 cytokine should work on the pathogenic process mainly provoked by Th1 inflammatory cytokines.

We found that after two or three exposures to malaria, mice developed antibody response. However, the antibody amounts drastically increased in 1-cure mice on day 7 PI more than the others (Fig.3A) but they almost die due to ECM, implying that parasite-specific antibodies at late stage could not prevent ECM in this mouse model study. The balance between consumption and production of antibody was maintained in 3-cure mice, presumably due to the suppression of IL-10 to Th2 cell activation, inhibiting the antibody production of plasma cells [89].

In our study, the role of IL-10 derived from CD19(+) cells, malaria specific antibodies and the mechanism how the donor CD19(+) cells interact with other cells in the recipient that develops primary malaria infection have not been confirmed. CD19(+) cells could be considered as an endogenous source of IL-10 in the recipient. Additionally, effector cells transformed from CD19(+) B cells is also able to play a protective role. Antigen specific antibodies and IL-10 secreting CD19(+) cells in semi-immune mice should be studied for inhibition of ECM. Similar transfer experiment of IL-10 producing B cells generated by non-pathogenic stimulus will determine their applicability in treatment of cerebral malaria.

Three-cure mice were also absolutely protected from ECM during the twelve and twenty months after the last challenge infection of malaria, demonstrating that the resistance against ECM could persist throughout life. This may explain why cerebral malaria occurs less commonly in adult and elderly people than in children in African endemic areas. Long term antibody production or T cell memory could prevent ECM in aged 3-cure mice. Previously, exposed children who had documented *P. falciparum* infections several years ago, but minimal exposure since then had similar frequencies of memory B cells to those of persistently malaria exposed children [90]. The chronic presence of the parasite might drive expansion of memory B cells in *P. falciparum*-exposed adults and children compared with naïve adults [91]. Therefore, memory B cells are one of candidates in suppressing ECM in aged 3-cure mice. Together, the expansion and long existence of malaria specific memory B cells in semi-immune mice is our expectation.

## Conclusion

Three times of repeated malaria infection were required for susceptible B6 mice to develop a complete resistance against ECM. The repeated malaria infection promoted the immunity against ECM in mouse model by splenic CD19(+) cells possibly via regulatory IL-10. The major source of IL-10 was CD5(−)CD19(+) cells (B1b). Further study will be performed to elucidate functional heterogeneity of semi-immune CD19(+) B cells in ECM. The protection against ECM in semi-immune mice was long lived despite of no further exposure to malaria. Studies targeting on human IL-10 producing B cells in people living in malaria-endemic regions should be conducted to clarify the whole spectrum of similar resistant mechanism in humans.

## Materials and Methods

### Mice, Parasites and establishment of experimental cerebral malaria

C57BL/6 (B6) mice were supplied by SLC laboratories, Fukuoka, Japan and were housed under the barrier conditions at the Animal Center of the Institute of Tropical Medicine. Mice used in all experiments were female and used between 6–8 weeks of age. *Plasmodium berghei* ANKA (*Pb*A) strain was originally obtained from T.Yanagi at National Bioresource Center at the Institute of Tropical Medicine Nagasaki University and used in all experiments after passaged once through naïve Balb/c mice. The *Pb*A was selected for its capacity of ECM induction in B6, with neurological signs (ataxia, paralysis, deviation of the head, and convulsion) appearing 6 to 10 days after infection [92]. Blood stages of the parasite were stored in liquid nitrogen at a concentration of 50% parasitized red blood cells in phosphate- buffered saline (PBS) containing 10% glycerol (Wako Pure Chemical Industries, Ltd, Japan). Cerebral malaria was induced by intra-peritoneal injection of 10^4^ *Pb*A-infected red blood cells (pRBCs). Parasitemia was monitored on Giemsa-stained thin blood smear from the day 1 to day 8 post infection (PI) and after that, every two days. The parasitemia was expressed as a percentage of more than 1000 RBCs. The day of patency was determined following microscopic examination of 10,000 RBCs.

### Generation of the semi-immune mice and challenge infection

The method based on a previous description [35] was pivotally modified. Briefly, C57BL/6 mice were infected with 10^4^ *Pb*A-pRBCs and then treated on day 5 after infection with intra-peritoneal injection of chloroquine (20 mg/kg/day) and pyrimethamine (20 mg/kg/day) daily for 7 days. Before subsequent rounds of infection, the parasite-clearance was confirmed and mice were rested for two weeks and then re-challenged with 10^4^ *Pb*A-pRBCs. Four groups of mice underwent zero (the control group without any infection and treatment), one, two, and three cycles of infection and treatment (designated as 0-cure, 1-cure, 2-cure and 3-cure) before finally challenged with 10^4^ *Pb*A-pRBCs without treatment. The course of infection was monitored twice daily for neurological symptoms including ruffled fur, abnormal postural responses, reduced reflexes and grip strength, coma, and convulsions. Mice that presented all these symptoms were considered as having ECM [25], [93]. The mice being either judged to have ECM or moribund to infection were euthanized. Mice were inhaled with lethal dose of diethyl ether (Wako Pure Chemical Industries, Ltd.) and then were exsanguinated via cardiac puncture. Three-cure mice were also intra-peritoneally challenged with 10^7^ of *Pb*A-pRBCs to observe high peripheral parasite, while 0-cure mice were inoculated with 10^4^ *Pb*A-pRBCs as the controls. The whole set of experiments were performed twice.

### Histopathological experiments

All animals were monitored closely for cerebral symptoms. Dead mice of 0-, 1-, 2-cure and sacrificed 3-cure mice were used for histopathological analysis. Brains were carefully removed after death and fixed by immersion in 10% paraformaldehyde in phosphate-buffered saline for at least 7 days. After fixation, preserved brain was transversally sectioned. These specimens were suspended in absolute alcohol, absolute xylene overnight, and then embedded on paraffin for one hour. Sections were cut to 5 µm thickness in four different positions: frontal lobe, temporal lobe, parietal lobe and occipital lobe, then stained with hematoxylin–eosin (HE) and analyzed by light microscopy. For each brain sample, all microscopic fields at 100×were examined for any histopathological changes including: blocked vessels, hemorrhages, edema perivascular cuffing and disruption and damage to cerebral vasculature endothelial linings [94]–[98].

### Plasma harvesting

On day 0, 3, 5 and 7 after challenge infection, four mice in each group of 0-cure, 1-cure, 2-cure and 3-cure were euthanized to collect blood samples via sterile cardiac puncture into tubes containing 5 UI of heparin as anticoagulant. Plasma was harvested after centrifugation of the whole blood. All plasma samples were stored in −80°C until further use for antibody and cytokine assays. Three-cure mice infected on day 7 after challenge infection and naïve mice were also euthanized to collect plasma for passive transfer experiment.

### Measurement of plasma *Pb*A-specific IgG and its subtype levels

The plasma levels of parasite-specific IgG and its isotype antibodies on day 0, 3, 5 and 7 PI of 0-cure, 1-cure, 2-cure and 3-cure B6 mice were estimated by ELISA as described previously [98]. Plasma was serially added in a dilution of 1/50–1/1,600 and a dilution was then chosen whereby all samples fell in the linear range of the curve; for all IgGs 1/100, dilution was applied. Briefly, the *Pb*A crude antigen was used at a protein concentration of 0.03 µg in 100 µl (0.3 µg/ml) of coating buffer (pH 9.6) per well to coat polystyrene plates (Lot number 091611, Nunc, Copenhagen, Denmark) and kept at 4°C overnight. The plates were washed thrice with 0.05% Tween-20-PBS. Optimum blocking conditions for non-specific binding was achieved using 200 µl per well of 0.1% blocking buffer (0.1% Tween-20/PBS, pH 7.2), and incubated for 1 hour at 37°C. Plates were washed thrice with PBS containing 0.05% Tween-20. The antigen in coated plates was then reacted with the plasma samples obtained from 0-cure, 1-cure, 2-cure and 3-cure mice at 1/100 dilutions, in duplicates. After two hours incubation at 37°C, plates were washed four times with 0.05% Tween-20/PBS. Later, 100 µl of diluted horse radish peroxidase (HRP)-conjugated goat anti-mouse IgG, IgG1 and IgG2a (Santa Cruz Biotechnology) was added to each well and incubated for 1 hour at 37°C, then washed thrice with 0.05% Tween-20/PBS. For color development, 3, 3′, 5, 5′-tetramethylbenzidine (TMB, stabilized chromate, Catalogue number 72673125A, Nitrogen, CA, USA) was used and prepared according to the manufacturer's instructions. The reaction was then interrupted after 15 minutes by the addition of 50 µl 1N H2SO4. Optical density (OD) was determined at 450 nm using EIA-reader (Bio-Rad, Hercules, CA).

### Plasma cytokine quantification

Determination of cytokine levels in the plasma of all mice was performed on day 0, 3, 5 and 7 PI. Cytokine (IFN-γ, IL-4, IL-10, and TGF-β) levels in plasma of all mice were measured using Procarta*®* Cytokine Assay Mouse Plex Kits (Affymetrix, Inc. Santa Clara, CA, USA) in duplicate, according to the manufacturer's instructions. The plate was read on a Luminex instrument, LABScan 100 (Luminex Corporation. Austin, USA). The detection threshold was 0.37 pg/ml. Data are presented as arithmetic means of duplicate values.

### Intracytoplasmic staining and flow cytometric analysis

The intracytoplasmic staining method was performed as in a previous studies [18], [69] with slight modification. Briefly, splenocytes were collected from 0-cure and 3-cure B6 mice on day 7 PI and re-suspended (10^6^ cells/ml) with parasitized erythrocytes (10^6^/ml) obtained from 0-cure mice (5–10% parasitemia) 7 days PI, lipopolysacharide (LPS)(10 mg/ml), phorbol 12-myristate 13-acetate (PMA) (50 ng/ml; Sigma), ionomycin (500 ng/ml; Sigma), and monensin (golgi plug, 2 mM; eBioscience) for 5 hours at 37°C. For IL-10 detection, Fc receptors were blocked with mouse Fc receptor-specific mAb (eBioscience) before cell-surface staining, then fixed and permeabilized with fixation/permeabilization solutions (Beckman Coulter) according to the manufacturer's instructions. Permeabilized cells were stained with PE-conjugated anti-mouse Foxp3 kit (eBioscience) and allophycocyanin-conjugated anti-mouse IL-10 (JES5-16E3; BDPharMingen). Data were acquired on a FACS Canto II flow cytometer (BD Biosciences) and analyzed using FlowJo software (Treestar, Ashland, OR, USA).

### Cell adoptive transfer experiments

Zero-cure and 3-cure mice were euthanized on day 7 PI for isolating splenic CD19(+) cells. Cells were sorted by using a magnetically activated cell sorting system (autoMACS, Miltenyi Biotec). Initially their spleens were homogenized in collagenase, re-suspended in PBS (pH 7.4). After incubation with 25 µl of anti-CD11c microbeads (Miltenyi Biotec), 225 µl of cell suspension from one spleen was first sorted for negative selection. CD11c(−)cell fraction was secondly incubated with 25 µl of anti-CD19 microbeads (Miltenyi Biotec) and then sorted into CD19 positive and negative population. The enriched CD19(+) cell fraction were isolated at >90% purity. On average, 3×10^7^ and 1.5×10^7^ of CD19(+) cells from one spleen of day 7-infected 3-cure and 0-cure mice, respectively, were harvested. Finally, 1.5×10^7^ (low dose) or 3×10^7^ (high dose) of CD19(+) cells of 0-cure or 3-cure mouse, re-suspended in total 250 µl of PBS, were injected intravenously (i.v.) into one naive mouse 24 hours before infection with 10^4^ of *Pb*A-pRBCs. PBS treated mice were used as controls.

### Statistical analysis

Differences in survival of semi-immune groups were analyzed using the log-rank test. Differences in parasitemia, antibody and cytokine levels were analyzed for statistical significance using the nonparametric Mann–Whitney U test. Values of parasitemia, antibodies and cytokines indicated in the text represent the median and inter-quartile range of the data. For all statistical tests, p<0.05 was considered as significance. The results of data were analyzed by GraphPad prism software (version 5.0).

## Supporting Information

Figure S1. **Representative photomicrograph (H&E staining) of the brain of an animal in each group on day 7 PI.** **A.** 0-cure mouse showing parenchymal microhaemorrhages in 2 places (arrows). **B.** 1-cure mouse showing parenchymal microhaemorrhages (arrow). **C.** 2-cure mouse showing parenchymal microhaemorrhages (arrow). **D.** 3-cure mouse not showing any parenchymal haemorrahage. Magnifications: **A**, **B**, **C** and **D** ×100
(TIF)
