# Supplementary figures and images for: CD19(+) B Cells Confer Protection against Experimental Cerebral Malaria in Semi-Immune Rodent Model
Source: PLoS One. 2013 May 28;8(5):e64836. doi: 10.1371/journal.pone.0064836 (PMC3665539; doi:10.1371/journal.pone.0064836)

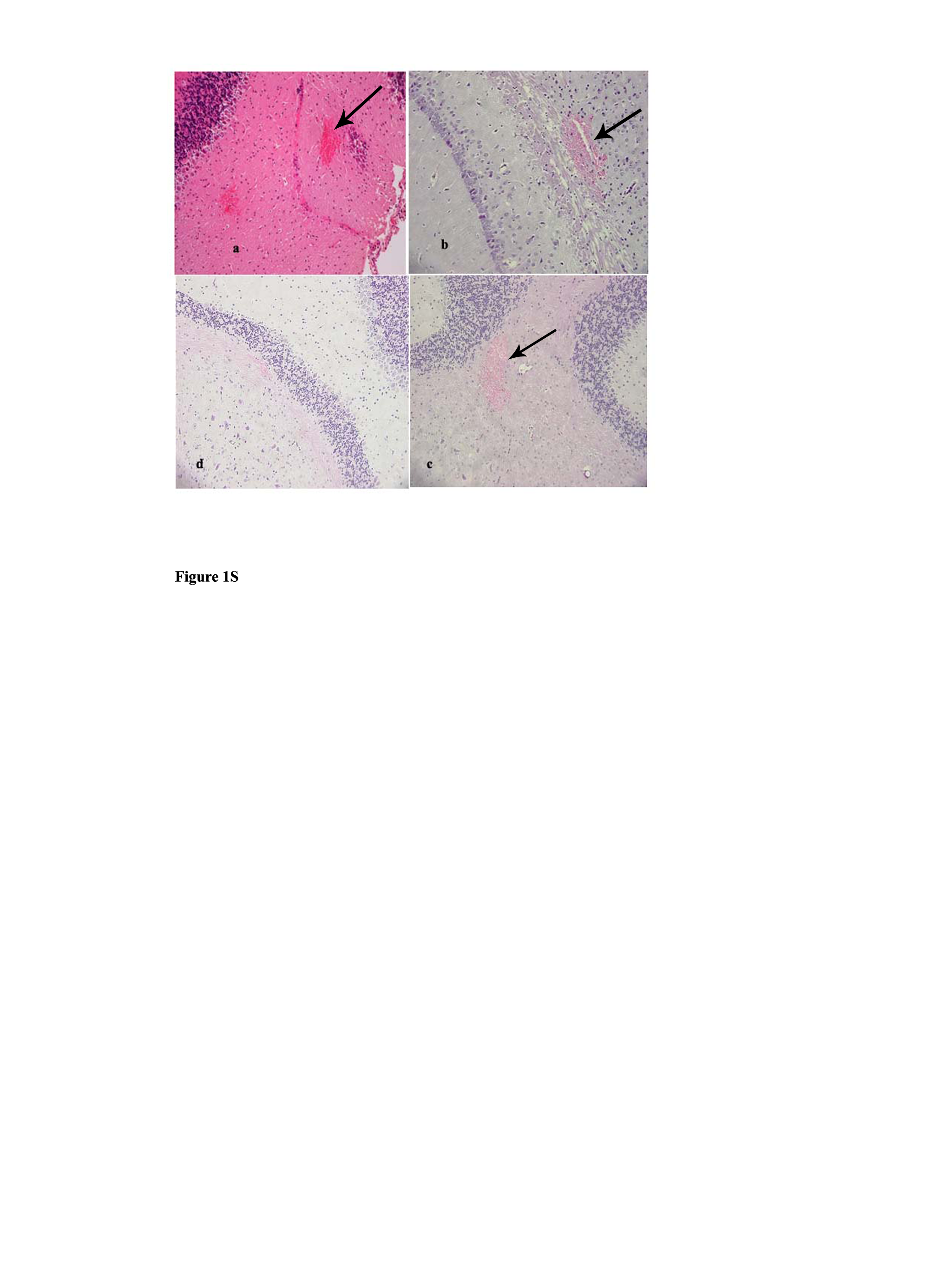

Supplement: Figure S1. — Representative photomicrograph (H&E staining) of the brain of an animal in each group on day 7 PI. A. 0-cure mouse showing parenchymal microhaemorrhages in 2 places (arrows). B. 1-cure mouse showing parenchymal microhaemorrhages (arrow). C. 2-cure mouse showing parenchymal microhaemorrhages (arrow). D. 3-cure mouse not showing any parenchymal haemorrahage. Magnifications: A, B, C and D ×100 (TIF) [file pone.0064836.s001.tif]
